# The Role of Pro-Inflammatory Chemokines CCL-1, 2, 4, and 5 in the Etiopathogenesis of Type 2 Diabetes Mellitus in Subjects from the Asir Region of Saudi Arabia: Correlation with Different Degrees of Obesity

**DOI:** 10.3390/jpm14070743

**Published:** 2024-07-11

**Authors:** Mohammad Muzaffar Mir, Jaber Alfaifi, Shahzada Khalid Sohail, Syeda Fatima Rizvi, Md Tanwir Akhtar, Mushabab Ayed Abdullah Alghamdi, Rashid Mir, Javed Iqbal Wani, Zia Ul Sabah, Fahad A. Alhumaydhi, Fahad Alremthi, AbdulElah Al Jarallah AlQahtani, Muffarah Hamid Alharthi, Masoud Ishag Elkhalifa Adam, Imadeldin Elfaki, Hany M. A. Sonpol

**Affiliations:** 1 Department of Basic Medical Sciences, College of Medicine, University of Bisha, Bisha 61922, Saudi Arabia; sksohail@ub.edu.sa (S.K.S.); sfatima@ub.edu.sa (S.F.R.); hany_sonpol@mans.edu.eg (H.M.A.S.); 2 Department of Child Health, College of Medicine, University of Bisha, Bisha 61922, Saudi Arabia; jalfaifi@ub.edu.sa; 3 Department of Public Health, College of Health Sciences, Saudi Electronic University, Riyadh 93499, Saudi Arabia; m.akhtar@seu.edu.sa; 4 Department of Internal Medicine, College of Medicine, University of Bisha, Bisha 61922, Saudi Arabia; mualghamdi@ub.edu.sa (M.A.A.A.); aaljarallah@ub.edu.sa (A.A.J.A.); 5 Prince Fahd Bin Sultan Research Chair, Department of MLT, Faculty of Applied Medical Sciences, University of Tabuk, Tabuk 71491, Saudi Arabia; rashid@ut.edu.sa; 6 Department of Internal Medicine, College of Medicine, King Khalid University, Abha 61421, Saudi Arabia; jwani@kku.edu.sa (J.I.W.); zsabah@kku.edu.sa (Z.U.S.); 7 Department of Medical Laboratories, College of Applied Medical Sciences, Qassim University, Buraydah 51452, Saudi Arabia; f.alhumaydhi@qu.edu.sa; 8 Diabetes and Endocrine Center, King Abdullah Hospital, Ministry of Health, Bisha 61922, Saudi Arabia; falshahrani28@moh.gov.sa; 9 Department of Family Medicine, College of Medicine, University of Bisha, Bisha 61922, Saudi Arabia; mualharthi@ub.edu.sa; 10 Department of Medical Education, College of Medicine, University of Bisha, Bisha 61922, Saudi Arabia; mieadam@ub.edu.sa; 11 Department of Biochemistry, Faculty of Science, University of Tabuk, Tabuk 71491, Saudi Arabia; ielfaki@ut.edu.sa

**Keywords:** type 2 diabetes mellitus, pro-inflammatory chemokines, obesity, CCL1, CCL2, CCL4, CCL5

## Abstract

Background: Type 2 diabetes mellitus (T2DM) is becoming a major global health concern, especially in developing nations. The high prevalence of obesity and related diabetes cases are attributed to rapid economic progress, physical inactivity, the consumption of high-calorie foods, and changing lifestyles. Objectives: We investigated the roles of pro-inflammatory chemokines CCL1, 2, 4, and 5 in T2DM with varying levels of obesity in the Asir region of Saudi Arabia. Materials and Methods: In total, 170 confirmed T2DM subjects and a normal control group were enrolled. Demographic data, serum levels of CCL-1, 2, 4, and 5, and biochemical indices were assessed in the subjects and control groups by standard procedures. Results: T2DM subjects were divided into four groups: A (normal body weight), B (overweight), C (obese), and D (highly obese). We observed that male and female control subjects had similar mean serum concentrations of pro-inflammatory chemokines CCL-1, 2, 4, and 5. T2DM subjects in all the four groups showed significantly higher levels of all the four chemokines compared to the controls, regardless of gender. In T2DM subjects with obesity and severe obesity, the rise was most significant. There was a progressive rise in the concentrations of CCL-1, 2, and 4 in T2DM subjects with increasing BMI. Serum CCL5 levels increased significantly in all T2DM subject groups. The increase in CCL5 was more predominant in normal-weight people, compared to overweight and obese T2DM subjects. Conclusions: Male and female control subjects had similar serum levels of pro-inflammatory chemokines CCL-1, 2, 4, and 5. The progressive rise in blood concentrations of three pro-inflammatory chemokines CCL-1, 2, and 4 in T2DM subjects with increasing BMI supports the idea that dyslipidemia and obesity contribute to chronic inflammation and insulin resistance. Serum CCL5 levels increased significantly in all T2DM subject groups. The selective and more pronounced increase in CCL5 in the T2DM group with normal BMI, compared to subjects with varying degrees of obesity, was rather surprising. Further research is needed to determine if CCL5 underexpression in overweight and obese T2DM subjects is due to some unexplained counterbalancing processes.

## 1. Introduction

Type 2 diabetes mellitus, also known as T2DM, is a complex disease that has rapidly become a major public health concern around the globe, particularly in less developed nations [1,2,3,4]. The rapid advancement of the economy, the rise in the number of people who are physically inactive, the consumption of foods high in calories, and the evolution of lifestyles are all factors that have contributed to the increased prevalence of obesity and diabetes [4,5]. As per the estimates of the International Diabetes Federation (IDF) regarding the prevalence of type 2 diabetes mellitus among adults around the world, it was anticipated that 536.6 million individuals were affected in 2021, which would account for 10.5% of the global population. In addition, IDF forecasts that by the year 2045, the number of people worldwide who have diabetes will have increased to 783.2 million, making up 12.2% of the total population [6]. China, the world’s second most populous developing nation, with a population that accounts for roughly one-fifth of the total global population, has seen a significant rise in the number of people diagnosed with diabetes in recent years [7]. As per recent estimates in Saudi Arabia, which has a total population of 24,194,300 people, the prevalence of diabetes is 17.7%, and there are around 4,274,100 adult diabetic subjects (https://idf.org/our-network/regions-members/middle-east-and-north-africa/members/46-saudi-arabia.html, accessed on 30 June 2024). Diabetes, which is consistently ranked among the top 10 causes of death around the world, can more than double or even triple a person’s likelihood of passing away from any cause [1,2]. Type 2 diabetes is responsible for nearly 95% of all cases of diabetes. The hallmark symptom is hyperglycemia, which lasts for an extended period of time and can be caused by either inadequate insulin synthesis or impaired insulin action [3,8].

It has been established that the frequency and occurrence of T2DM varies greatly around the world based on ethnicity and geographic location, with the largest risks being faced by Japanese, Hispanic, and native American individuals [9,10,11,12]. The incidence rates of T2DM among Asians have been observed to be higher compared to white American and UK populations [13]. It is noteworthy that the black population is the category with the highest risk level in both demographics [1].

Although the precise etiology has not yet been determined, there are a number of factors that have been proposed as possible contributors [14,15]. These include social and economic variables, innate genetic predispositions, interactions between genes and the environment, and current lifestyle elements that contribute to obesity. A person’s genetic susceptibility has a profound bearing on the likelihood that they may develop T2DM. The complex polygenic aspects of T2DM have been revealed, thanks to the numerous genome-wide association studies that have been carried out over the course of the past decade [4,14,15]. By primarily influencing the body’s ability to produce insulin, the great majority of these genetic loci raise the chance of developing T2DM.

Long-term risks of T2DM include kidney failure, blindness, and cardiovascular disease, in addition to diabetic neuropathy. Acute problems include diabetic ketoacidosis and hyperosmolar hyperglycemic syndrome [16]. Insulin resistance (IR), metabolic syndrome (MS), and decreased insulin production are hallmarks of T2DM; these factors contribute to the pathophysiology of the disease and make it practically impossible to effectively manage glycemic levels. It is estimated that 90 percent of people who have diabetes are either overweight or obese, and obesity has emerged as one of the most significant risk factors for T2DM [17,18].

Recent observations suggest that inflammation, immunological dysregulation, alterations in gut microbiota, and cytokine dysregulation are the key pathophysiological contributors to T2DM [3,4,8,14]. The focus of many recent investigations has been on unraveling the molecular bases of metabolic inflammation and its association with cardiometabolic disease and T2DM [19,20]. This inflammatory nexus is a hallmark of obesity and MS [21,22]. Obesity-induced inflammation is mainly mediated by immune cells, especially macrophages and T lymphocytes [22,23]. In particular, adipose tissue macrophages (ATMs) are major sources of pro-inflammatory cytokines, which participate in diverse molecular signals leading to IR [23].

Chemokines, a well-known family of cytokines, play a significant role in inflammation and autoimmune disorders [23,24]. More than 50 chemokines and a set of around 20 chemokine receptors expressed in adipocytes have been implicated in the acute and chronic inflammatory processes [25,26,27]. Chemokines can be broken down into four groups, each based on a different motif sequence containing two N-terminal cysteine residues (where X is any amino acid residue) [27]. Most CC chemokines act on monocytes, T cells, eosinophils, and basophils, which mediate chronic inflammation and allergies, while CXC chemokines are mostly chemotactic for neutrophils and are known to be involved in acute inflammation. Complex metabolic signals involving obesity and inflammation involve chemokines, since most chemokines bind to multiple chemokine receptors, and chemokine receptors have overlapping ligand specificities [22,25,27].

Chemokines are expressed in response to diverse stimulations, including excess nutrients, and lead to an inflammatory cascade, which creates a favorable ambiance for obesity, the dysfunction of beta cells, and/or reduced insulin production [25,28]. Many pro-inflammatory chemokines have been implicated in the pathogenesis of T2DM, which is supposed to be multifaceted [22,24].

CCL1: Chemokine (C-C motif) ligand 1 is a small inducible glycoprotein cytokine, and is also known as I-309 or TCA-3. It is encoded by the gene Scya 1, and binds CCR8 as its receptor [22,24]. Increased levels of CCL1 have been reported in T2DM subjects with comorbidities and diabetic complications [22,29]. The reports are inconsistent between investigators, with some suggesting a role of CCL1 in the pathogenesis of T2DM, and others holding a contrary opinion [24,29]. Low sample sizes have also been a common feature of these studies, thus justifying the further investigation of CCL1 in T2DM subjects with a reasonable sample size.

CCL2: CCL2 is commonly known as monocyte chemoattractant protein-1 (MCP-1). MCP-1 is an established pro-inflammatory cytokine due to its chemotactic activity. It has been implicated in the pathogenesis of many diseases, including atherosclerosis, obesity, and type 1 diabetes mellitus (T1DM) [30,31]. High levels of CCL2 have been reported to be protective against T1DM, and intriguingly associated with its complications [30,31]. As many studies have shown high CCL2 levels in T2DM [22]. CCL2 is involved in the recruitment and differentiation of macrophages to a pro-inflammatory state [30,32]. Several studies have shown positive outcomes in diabetic complications in association with decreased levels of CCL2 [32].

CCL4: CCL4 is a pro-inflammatory chemokine, and is also known as macrophage inflammatory protein-beta (MIP-1β). It is encoded by the gene Scya4 and signals via the receptors CCR1 and CCR5. CCL4 has been reported to be upregulated in both type 1 and 2 diabetes, and the circulatory levels of CCL4 are inversely associated with proinsulin. [22,33]. The inhibition of CCL4 has been reported to improve IR and decrease the chances of a hyperglycemic state leading to the progression of T2DM. [33].

CCL5: As a pro-inflammatory chemokine, CCL5 helps in the recruitment of leukocytes to the site of inflammation. Also known as RANTES (regulated on activation, normal T cell expressed and secreted), it is an 8 kDa protein made up of 68 amino acids, and is encoded by the Scya5 gene [34]. CCL5 is associated with many diseases, including cardiovascular disorders, cancer, and different types of diabetes, including T2DM [30,34,35]. CCL5 is thought to be associated with IR in relation to age, HbA1c, obesity, and other factors, although the number of studies is limited [36]. With this background, we intend to study the relationship between pro-inflammatory chemokines CCL1, CCL2, CCL4, and CCL5 and the etiopathogenesis of T2DM in subjects with varying levels of obesity, BMI, and HbA1c in a patient cohort from the Asir region of Saudia Arabia. This is an ongoing study, and we are recruiting more subjects.

## 2. Methods

**Figure 1:**
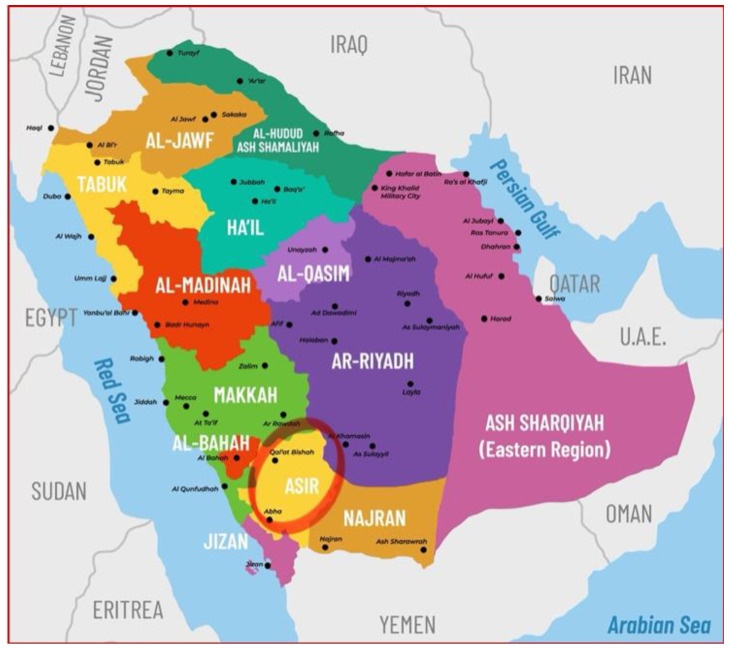
Map showing different provinces of Saudi Arabia. The study areas (Asir region) is encircled in red.

Study population: This collaborative case–control study was conducted on 200 T2DM subjects and 100 healthy controls from the Asir region of Saudi Arabia (Figure 1).

All the study participants were Saudi citizens. The blood samples were collected at the Diabetic Centre, King Abdullah Hospital (KAH) in Bisha, and the Asir General Hospital (AGH) in Abha. The recruitment period of the T2DM subjects and controls was from March 2020 to May 2022. Informed consent was obtained from all T2DM and control subjects before the collection of blood samples.

### 2.1. Ethical Statement

Ethical approval was obtained from the local RELOC Committee of the College of Medicine, University of Bisha (Ref. No. UB-RELOC H-06-BH-087/ (0504 of 23), dated 1 June 2023), in accordance with the local guidelines, which conform, in essence, to the principles of the Helsinki Declaration.

### 2.2. Inclusion Criteria

Only Saudi citizens living in the Asir region (Bisha and Abha cities and surrounding areas) were enrolled in this study. In total, 200 confirmed T2DM subjects who were on oral hypoglycemic agents and/or insulin were included in this study (110 males and 90 females).

### 2.3. Exclusion Criteria

T2DM subjects with other significant chronic diseases or malignancies were excluded from the study. Type 1 diabetes subjects were also excluded from the study.

Inclusion criteria for controls: In total, 100 control subjects were initially enrolled. These were healthy volunteers with no history of diabetes or any major clinical disorders, and with normal fasting and random plasma glucose levels.

Exclusion criteria for controls: Control subjects who had abnormal laboratory indices were excluded from the study. As a result, 85 control subjects (44 males and 41 females) were included in the study. The other criterion for selecting this limited number of control subjects was the availability of ELISA kits, as each 96-well kit is sufficient for less than 88 samples after including calibrators and QC samples.

Data collection: Finally, 170 Saudi citizens with confirmed T2DM (96 males and 74 females) who visited KAH and AGH for regular follow-ups and met the inclusion criteria were included in the study. T2DM was diagnosed according to the parameters of the WHO criteria. Case history, age, gender, body mass index (BMI), glycated hemoglobin (HbA1c), fasting and random blood glucose levels, total cholesterol, triacylglycerol (TG), high-density lipoprotein cholesterol (HDL-C), and low-density lipoprotein cholesterol (LDL-C) concentrations were among the various variables analyzed from the T2DM subjects and controls. Established techniques were used to measure the anthropometric and biochemical parameters.

Blood specimen collection from T2DM: For each T2DM patient, 4 mL of peripheral blood was drawn into a red-top tube, without the use of any anticoagulants, after an overnight fast. One serum aliquot was immediately stored at −20 °C until the estimation of chemokines. The second serum sample was sent away immediately for biochemical evaluation.

Blood specimen collection from healthy controls: Fasting blood sample collections from all healthy age-matched control subjects were timed around routine blood draws that were part of standard health examinations. This eliminated the need for additional phlebotomies. For all controls, a sample of peripheral blood measuring about 4 mL was taken and placed in a red-top tube without any anticoagulant. One serum aliquot was immediately stored at −20 °C until the estimation of chemokines. The second serum part was immediately used for biochemical evaluation.

Estimation of biochemical parameters: All serum chemistry investigations were performed on a random-access, multi-channel analyzer (Roche Diagnostics) in the medical laboratory department at KAH, Bisha. Commercially available test kits including calibrators and internal quality control samples from the manufacturer were used for these analyses.

Estimation of chemokines: The serum concentrations of the four pro-inflammatory chemokines, viz., CCL1, CCL2, CCL4, and CCL5, were determined by enzyme-linked immunosorbent assays (ELISA) using commercially available kits from Abcam, UK. The intra- and inter-assay variations were less than 5%.

CCL1: The serum level of CCL1 was determined using a commercially available, high-sensitivity CCL1 human ELISA Kit (Abcam, Cambrige, UK, Cat No. ab314600, 96 wells) with a sensitivity of 1.4 pg/mL and a measuring range of 4.7–300 pg/mL. The assay was performed as per the instructions given by the manufacturer and the results are reported as pg/mL.

CCL2 (MCP-1): The serum level of CCL2 was determined using a commercially available human MCP-1 ELISA kit (Abcam, Cambrige, UK, Cat No. ab179886) with a sensitivity of 1.26 pg/mL and a measuring range of 4.7–300 pg/mL. The assay was performed as per the instructions given by the manufacturer and the results are reported as pg/mL.

CCL4 (MIP-β): The serum level of CCL4 was determined using a commercially available human MIP-β ELISA kit (Abcam, Cambrige, UK, Cat No. Ab100597) with a sensitivity of 2.5 pg/mL and a measuring range of 4.1–1000 pg/mL. The assay was performed as per the instructions given by the manufacturer and the results are reported as pg/mL.

CCL5 (RANTES): The serum level of CCL5 was determined using a commercially available human RANTES ELISA kit (Abcam, Cambrige, UK, Cat No. ab174446) with a sensitivity of 0.091 pg/mL and a measuring range of 0.94–60 pg/mL. The assay was performed as per the instructions given by the manufacturer and the results are reported as pg/mL.

Statistical analysis: SPSS, version 20, was used to conduct the statistical analysis. Data that had a normally distributed distribution are presented as means with standard deviations (SD), while data that had a skewed distribution are displayed as medians (Q1–Q3). One-way analysis of variance (ANOVA) with the Tukey HSD test was used to determine the significance of differences for variables with normal distribution and homogenous variances; otherwise, Kruskal–Wallis one-way analysis of variance by rank and the multiple-comparison post hoc test were used. Values equal to or less than 0.05 (*p* < 0.05) were deemed significant. The multivariate correlation technique was applied to explore the association between various variables in the control and T2DM subjects. Shapiro–Wilk and Mardia’s tests were used to examine the normality of the univariate and multivariate data, respectively. In addition, density plots and normal Q-Q plots were used for different variables to assess the data’s normality.

## 3. Results

**Figure 2:**
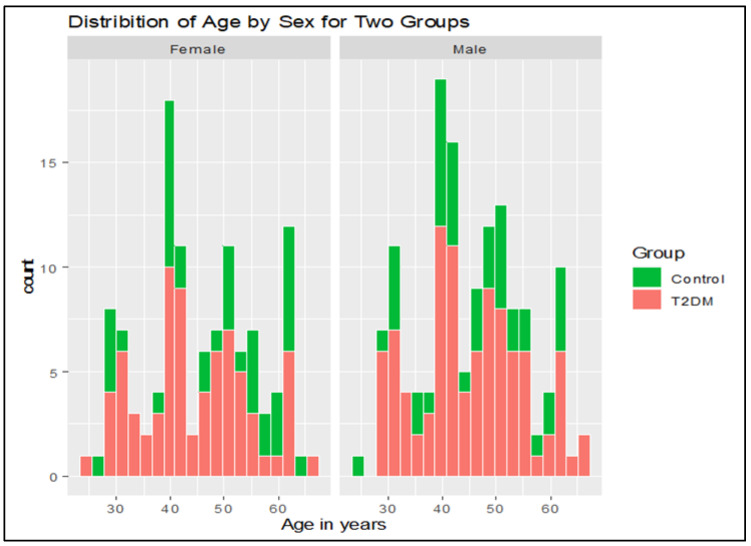
Histogram showing the distribution of age between controls (green) and T2DM subjects (red) across two genders.

Anthropometric indices and biochemical parameters of male T2DM subjects: Table 1 lists the anthropometric and biochemical indices of the male T2DM subjects. The 96 male T2DM subjects were divided into four groups on the basis of their BMI values. Group A comprised 25 T2DM subjects with a normal BMI of 21.80 ± 2.14 kg/m^2^; Group B included 23 overweight subjects with a BMI of 27.44 ± 2.29 kg/m^2^; Group C had 24 subjects with obesity and a BMI of 34.94 ± 3.18 kg/m^2^; and Group D included 24 T2DM subjects with severe obesity and a BMI of 47.22 ± 5.70 kg/m^2^. The 44 male control subjects had a normal BMI of 21.74 ± 1.78 (mean ± SD) kg/m^2^, as is presented in Table 1. The age for male T2DM subjects ranged from 28 to 67 years (mean of 46.5 ± 1.02), and for controls from 28 to 62 years (mean of 46 ± 0.86). The distribution of age between the control and T2DM subjects across the two genders is shown in Figure 2.

**Figure 3:**
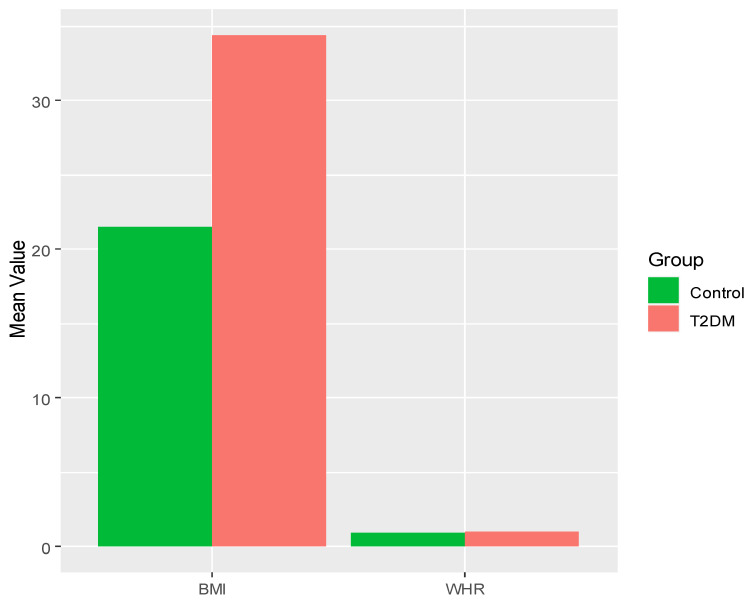
Bar plot displaying the mean values of WHR and BMI across the T2DM subjects and control group. T2DM subjects in Group C had significantly higher BMI compared to Groups A and B with *p* < 0.01, whereas Group D subjects had very significantly increased BMI versus Groups A and B with *p* < 0.001, and also significantly increased BMI versus Group C with *p* < 0.01 (Figure 3).

There was no significant difference in WHR in Groups A and B compared to the controls, while the Group C and Group D subjects showed highly significant changes in WHR compared to the controls. Group D showed a very significant increase in WHR *p* < 0.001 compared to Groups A and B, whereas Group C subjects showed a significant increase (*p* < 0.01) in WHR versus Groups A and B. The T2DM subjects in Groups A and B did not exhibit any significant increase in BMI compared to the controls, whereas BMI increased significantly in Groups C and D compared to the controls. A bar plot displaying the mean value of WHR and BMI across the different groups is depicted in Figure 3.

The T2DM subjects in Groups A, B, and C did not show any significant differences in fasting glucose levels compared to the controls, whereas the Group D subjects had significantly higher fasting glucose values (*p* < 0.001). HbA1c did not exhibit any significant differences among the various patient groups (Groups A, B, and D), although it was considerably higher in all four groups of T2DM subjects when compared to the control group, with *p* < 0.001. The T2DM subjects in Group C showed significantly higher HbA1c levels compared to Groups A, B, and D (*p* < 0.001). Groups A to C depicted significant differences in total cholesterol in comparison with the control group (*p* < 0.01), but Group D showed a very significantly increased total cholesterol level compared to the controls (*p* < 0.001). Groups A–C did not show any significant differences in the total cholesterol levels mutually. HDL cholesterol levels were unremarkable in all the subjects compared to the control group, although there was an apparent and comparable decrease in HDL values in the patient groups. LDL cholesterol was similar in control subjects and T2DM subjects in Group A, but was significantly higher in patient Groups B and C compared to the controls (*p* < 0.001). Group D subjects also had significantly higher LDL levels compared to the Group A T2DM subjects and the controls (*p* < 0.001). The triglyceride levels in the T2DM subjects of Groups A–C were significantly higher compared to the controls (*p* < 0.01). The Group D subjects showed a very significant increase in TG levels with *p* < 0.001 in comparison to the controls. Group D also reported significantly higher TG levels compared to the T2DM subjects in Groups A–C with *p* < 0.01.

Anthropometric indices and biochemical parameters of female T2DM subjects: Table 2 summarizes the anthropometric and biochemical indices of the 74 female T2DM subjects. As can be seen in Table 2, the age of the 74 female T2DM subjects ranged from 27 to 69 years (mean of 46.25 ± 0.99), and for the controls from 27 to 64 years (mean of 48 ± 0.85). WHR was significantly higher in Group C and Group D subjects compared to the control subjects. Group D showed a very significant increase in WHR (*p* < 0.001) compared to the controls and Groups A and B, whereas Group C subjects showed a significant increase in WHR versus Groups A and B and the control group with *p* < 0.01.

The 41 control subjects in the female group had a normal BMI of 21.20 ± 1.66 kg/m^2^. Based on their BMI values, the female T2DM subjects were divided into four groups. Group A comprised 18 subjects with normal body weight and a BMI of 21.41 ± 2.14 kg/m^2^; Group B included 19 overweight subjects with a BMI of 28.22 ± 2.26 kg/m^2^; Group C had 20 subjects with obesity and a BMI of 33.88 ± 3.20 kg/m^2^; and Group D included 17 T2DM patients with severe obesity and a BMI of 46.48 ± 5.20 kg/m^2^. BMI showed no significant increase in Groups A and B, whereas it was very significantly increased in Groups C and D compared to the controls (*p* < 0.001). Group C subjects had significantly increased BMI compared to Groups A and B with *p* < 0.01, whereas Group D showed a highly significant increase in BMI versus Groups A and B (*p* < 0.001) and a significantly increased BMI versus Group C with *p* < 0.01.

The fasting glucose values in Groups A to C were significantly higher compared to the controls with *p* < 0.01, while Group D had significantly higher fasting glucose concentrations compared with the controls *p* < 0.001. HbA1c was very significantly increased in all four female T2DM patient groups compared to the control group with *p* < 0.001, but did not show any significant difference among the different patient groups (A–D) mutually.

**Figure 4:**
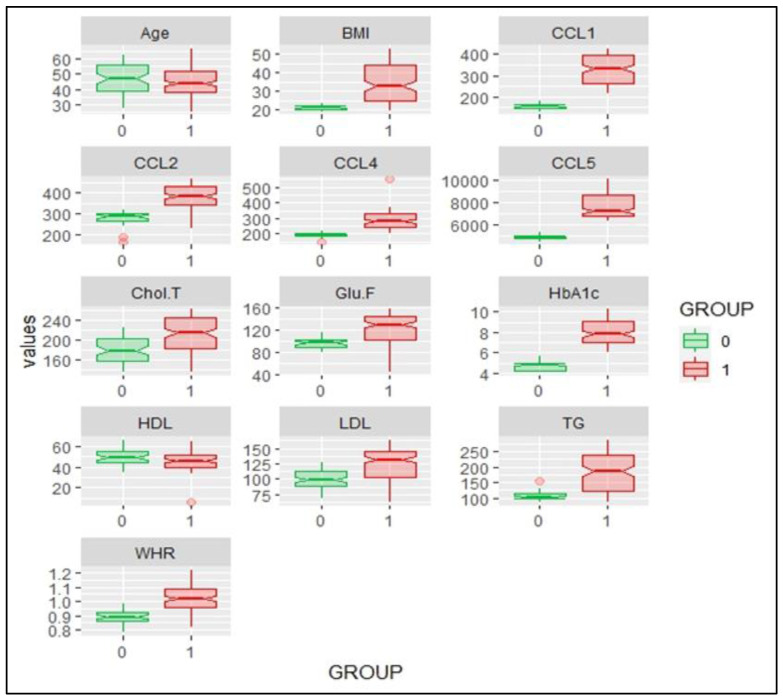
Box plot showing anthropometric indices, biochemical parameters, and chemokine levels of control and T2DM subjects; 0 in green represents controls and 1 in red represents T2DM subjects.

The female T2DM patient Groups A and B showed significantly higher total cholesterol values compared to the control subjects with *p* < 0.01. The Group C and D subjects showed significantly higher levels of total cholesterol compared to the controls with *p* < 0.001. The T2DM subjects in Groups C and D also depicted significantly higher total cholesterol results compared to Groups A and B with *p* < 0.01. HDL cholesterol levels remained similar in Groups A and B compared to the control group, but showed an insignificant decrease in Groups C and D when compared to the controls. LDL cholesterol was similar in the controls and the Group A T2DM subjects, but was significantly higher in patient Groups C and D compared to the controls (*p* < 0.001). Group D subjects depicted significantly higher LDL levels compared to the Group A T2DM subjects (*p* < 0.001). The triglyceride levels in the T2DM subjects of Groups A–C were significantly higher than the controls (*p* < 0.01). The Group D subjects showed a very significant increase in TG levels with *p* < 0.001 in comparison to the controls. Group D also reported significantly higher TG levels compared to Groups A and B with *p* < 0.01. The summary statistics of the anthropometric indices, biochemical parameters, and chemokine levels of the control and T2DM subjects are depicted in Table 3. The distributions of the different variables in the control and T2DM subjects are summarized in the box plot in Figure 4.

### 3.1. Chemokine Concentrations in Male T2DM Subjects and Controls

The serum levels of chemokines CCL1, CCL2, CCL4, and CCL5 in the male T2DM subjects and control subjects in Groups A to D are depicted in Table 4.

CCL1 (I-309 or TCA-3): The average serum level of CCL1 was 158 ± 21.02 pg/mL in the normal male controls. CCL1 levels were significantly elevated in patients in Groups A and B compared to the controls with *p* < 0.01. Groups C and D depicted elevated levels of CCL1 with very high significance (*p* < 0.001) compared to the control subjects. Severely obese T2DM subjects in Group D showed higher levels of CCL1 compared to Group C with *p* < 0.04. Group D T2DM subjects also showed significantly high levels of CCL1 compared to Groups A and B, with *p* < 0.01. Obese T2DM subjects in Group C showed significantly elevated levels of CCL1 compared to the Group A T2DM subjects with normal body weight.

CCL2 (MCP-1): The mean serum level of CCL2 was 288 ± 33.12 pg/mL in the normal male controls. CCL2 levels were significantly increased in patient Groups A and B compared to the controls with *p* < 0.05. Obese and highly obese subjects in Groups C and D depicted elevated levels of CCL2 with very high significance (*p* < 0.001) compared to the control subjects. Severely obese T2DM subjects in Group D showed higher levels of CCL2 compared to Group C with *p* < 0.04. Group D T2DM subjects also showed significantly high levels of CCL2 compared to Groups A and B, with *p* < 0.01. Obese T2DM subjects in Group C showed significantly elevated levels of CCL2 compared to Group A T2DM subjects with normal body weight, with *p* < 0.01.

CCL4 (MIP-1β): The mean serum level of CCL4 was 186 ± 22.56 pg/mL in the normal male controls. CCL4 levels were significantly increased in Groups A and B compared to the controls with *p* < 0.05. Obese and highly obese subjects in Groups C and D depicted elevated levels of CCL4 with very high significance (*p* < 0.001) compared to the control subjects. Severely obese T2DM subjects in Group D showed higher levels of CCL4 compared to Group C with *p* < 0.04, and significantly higher levels of CCL4 compared to Groups A and B with *p* < 0.01.

### 3.2. CCL5 (RANTES)

**Figure 5:**
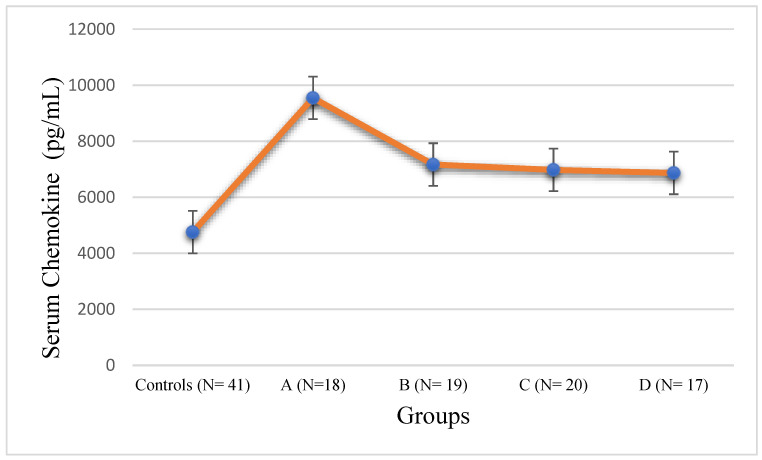
Error diagram of chemokine CCL5 levels in male controls and T2DM subjects.

The mean serum level of CCL5 was 4880 ± 348.98 pg/mL in the normal male controls. The increase in CCL5 levels in Group A was very highly significant compared to controls, with *p* < 0.001. Groups B and C showed significantly elevated levels of CCL5 compared to the controls with *p* < 0.01. Groups B, C, and D also showed significantly decreased levels of CCL5 compared to Group A with *p* < 0.01. Severely obese T2DM subjects in Group D showed higher levels of CCL5 compared to the controls with *p* < 0.05, and significantly lower levels of CCL5 compared to the T2DM subjects with normal body weight with *p* < 0.01. The error diagram of serum CCL5 levels in T2DM subjects and male controls is displayed in Figure 5.

### 3.3. Chemokine Concentrations in Female T2DM Subjects and Controls

The serum levels of chemokines CCL1, CCL2, CCL4, and CCL5 in the female T2DM subjects and control subjects are summarized in Table 5.

CCL1 (I-309 or TCA-3): The mean serum level of CCL1 was 152 ± 20.88 pg/mL in the normal female controls. CCL1 levels were significantly elevated in Groups A and B (262 ± 27.52 and 311 ± 25.10 pg/mL, respectively) compared to the controls, with 152 ± 20.88 pg/mL (*p* < 0.01). Obese and very obese female subjects in Groups C and D depicted elevated levels of CCL1 with very high significance (*p* < 0.001) compared to the control subjects. Severely obese female T2DM subjects in Group D showed higher levels of CCL1 compared to Group C with *p* < 0.04. Group D T2DM subjects also showed significantly high levels of CCL1 compared to Groups A and B, with *p* < 0.01. Obese female T2DM subjects in Group C showed significantly elevated levels of CCL1 compared to Group A T2DM subjects with normal body weight.

CCL2 (MCP-1): The mean serum level of CCL2 was 275 ± 32.14 pg/mL in normal female controls, which is more or less similar to that of the male control subjects. CCL2 levels were significantly increased in Groups A and B compared to the controls with *p* < 0.05. Obese and highly obese subjects in Groups C and D depicted elevated levels of CCL2 with very high significance (*p* < 0.001) compared to the control subjects. Severely obese female T2DM subjects in Group D showed higher levels of CCL2 compared to Group C, with *p* < 0.04. Group D T2DM subjects also showed significantly high levels of CCL2 compared to Groups A and B, with *p* < 0.01. Obese T2DM female subjects in Group C showed significantly elevated levels of CCL2 compared to Group A T2DM subjects with normal body weight, with *p* < 0.01.

CCL4 (MIP-1β): The mean serum level of CCL4 in the female control subjects was 194 ± 22.54 mg/mL, compared to 186 ± 22.56 pg/mL in the male controls. CCL4 levels were significantly increased in Groups A and B compared to the controls with *p* < 0.05. Obese and highly obese subjects in Groups C and D depicted elevated levels of CCL4 with very high significance (*p* < 0.001) compared to the control subjects. Obese T2DM female subjects in Group C showed significantly elevated levels of CCL2 compared to Group A and B T2DM subjects with *p* < 0.04. Severely obese T2DM subjects in Group D showed higher levels of CCL4 compared to the obese Group C T2DM subjects with *p* < 0.04, and significantly high levels of CCL4 compared to Groups A and B with *p* < 0.01.

### 3.4. CCL5 (RANTES)

**Figure 6:**
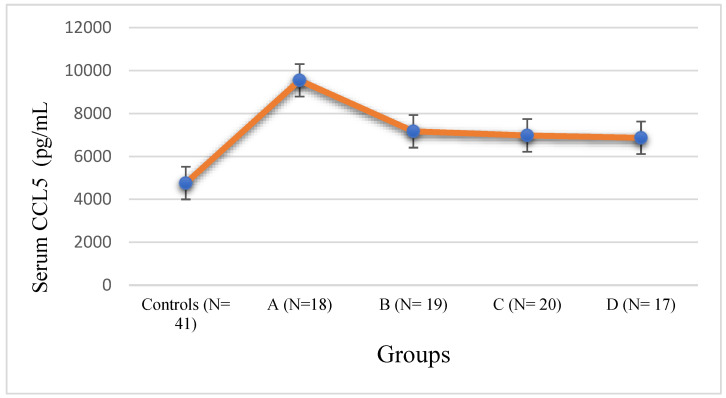
Error diagram of chemokine CCL5 levels in female controls and T2DM subjects.

The mean serum level of CCL5 in the female control subjects was 4756 ± 342.56 pg/mL, compared to 4880 ± 348.98 pg/mL in the male controls. The mean CCL5 level in Group A (9546 ± 522.44 pg/mL) was very highly elevated compared to the controls with *p* < 0.001. Groups B and C showed significantly elevated levels of CCL5 compared to the controls with *p* < 0.01. Groups B, C, and D also showed significantly decreased levels of CCL5 compared to Group A, with *p* < 0.01. Severely obese T2DM subjects in Group D showed higher levels of CCL5 compared to the controls with *p* < 0.05, and significantly lower levels of CCL5 compared to the T2DM subjects with normal body weight, with *p* < 0.01. The error diagram of serum CCL5 levels for the female T2DM subjects and controls is displayed in Figure 6.

**Figure 7:**
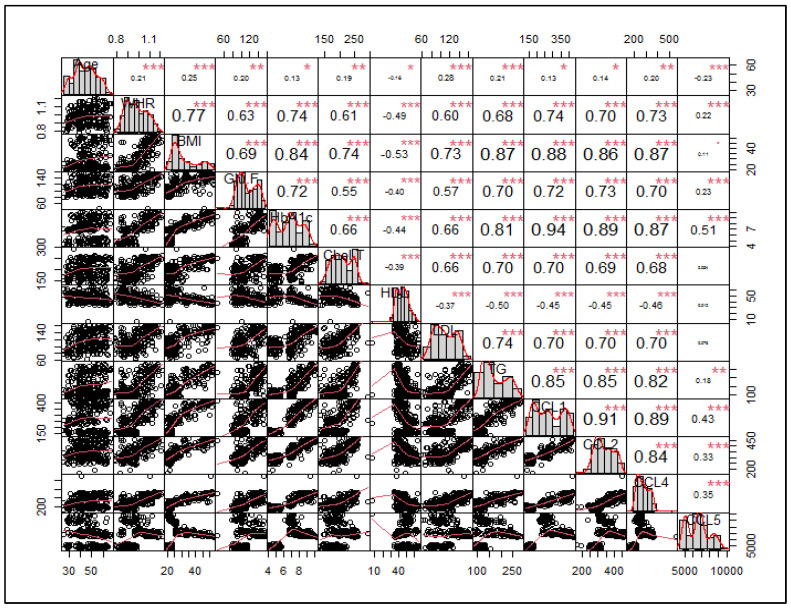
Correlation plot of different variables in control and T2DM subjects. *** represents highly significant, ** represents significant, and * is significant but close to 0.05.

Multivariate correlation analysis: The multivariate comparisons were performed by logistic regression analysis, in which controls and T2DM patient groups were compared with respect to all the variables. The correlation analysis of anthropometric indices, biochemical parameters, and chemokine levels in the control and T2DM subjects is depicted in Figure 7. As is evident, all the variables were correlated to different levels of significance, except CCL 5, which showed a lack of association with parameters like BMI, Chol-T, HDL, LDL, etc.

**Figure 8:**
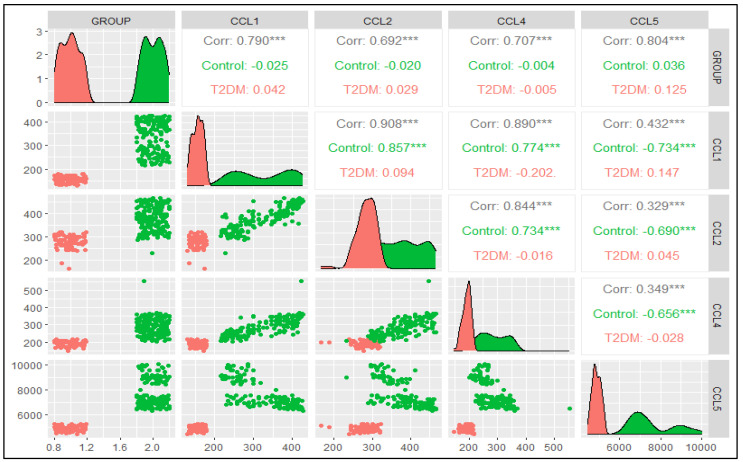
Correlation plot of four chemokines in control and T2DM subjects. *** represents highly significant correlation.

Correlation analysis for chemokines: The four chemokines, CCL1, CCL2, CCL 4, and CCL 5, were analyzed in the control and T2DM subjects by linear regression, and the results are presented in Figure 8.

## 4. Discussion

Insulin resistance and faulty insulin production are hallmarks of the pathogenesis of T2DM. One of the most prominent risk factors for type 2 diabetes is obesity [8,14,15]. Several studies have revealed key pathophysiological aspects of T2DM, including inflammation, chemokine dysregulation, gut microbiome alterations, and immunological dysregulation [3,4,8,14]. Many inflammatory cytokines and other variables contribute to the inflammatory nexus that is characteristic of obesity and MS [19,23,24]. Pro-inflammatory chemokines have been linked to the etiology of T2DM, although the results have been mixed and inconsistent [22,23,24]. Using a T2DM and a control cohort from the Asir region of Saudia Arabia, we looked into the association between the pro-inflammatory chemokines CCL1, CCL2, CCL4, and CCL5 and the etiopathogenesis of T2DM in subjects with different degrees of obesity, BMI, and HbA1c.

CCL1 (I-309 or TCA-3): The mean levels of CCL1 were similar in male (N = 44) and female (N = 41) control subjects, which is in agreement with previous studies [22,24,29]. CCL1 levels in T2DM subjects with normal body weight (Group A) and in overweight subjects (Group B) were significantly higher than in the male and female controls, with *p* < 0.01. Obese and highly obese T2DM subjects of both genders in Groups C and D depicted very highly significant elevations in CCL1 levels with *p* < 0.001. The highest levels of CCL1 were observed in Group D. These results are in conformity with earlier studies [22,24]. CCL 1 is believed to mediate the recruitment of monocytes, macrophages, Th2, and Treg cells by interacting with the CCR8 chemokine receptor [22,37]. This turn of events results in the release of many pro-inflammatory cytokines, including IL-1 β [22,38]. The resultant inflammatory cascade results in the impaired secretion of insulin in β islet cells [39]. IL-6 and IL-1 β are involved in IR, which is mediated through the underexpression of insulin receptor substrate-1 (IRS-1), and also suppress the activity of lipoprotein lipase, resulting in hypertriglyceridemia [40]. CCL1 is believed to have the dual purpose of predisposing subjects to both obesity and insulin resistance; this assumption seems plausible in light of the significantly higher levels of CCL1 in T2DM subjects in all four groups, A to D, ranging from normal body weight to severe obesity.

CCL2 (MCP-1): CCL-2 is a pro-inflammatory chemokine that exerts its action by specifically binding to CCR2 receptors. The mean levels of CCL2 were similar in male (288 ± 33.12 pg/mL) and female (275 ± 32.14 pg/mL) controls, which agrees with previous studies [2,3,8]. CCL2 levels in T2DM subjects with normal body weight (Group A) and in overweight subjects (Group B) were significantly higher than in the male and female controls, with *p* < 0.05. Obese and highly obese T2DM subjects of both genders (Groups C and D) showed very highly significant elevations in CCL2 levels with *p* < 0.001. Group C (obese T2DM subjects) showed significantly elevated levels of CCL2 compared to the controls, with *p* < 0.01. Although a few studies reported lower levels of CCL-2 in the prediabetic state, our results are partly consistent with earlier studies that reported higher levels of CCL2 in prediabetes, T1DM, and T2DM in general, as there was no further subdivision on the basis of BMI and level of obesity in those studies [2,3,8,22]. A previous study from KSA showed higher levels of CCL2 in obese women and lower levels in obese men, without any significance, which contrasts our study outcomes and previous studies [41]. Overnutrition and resultant obesity is involved in the activation of adipose tissue, especially ATMs, leading to higher levels of CCL2 [24,42]. CCL2 plays an active role in the recruitment of monocytes, NK cells, and other inflammatory cells, and hence is a significant contributor to the inflammatory cascade, which is a turning point in IR and MS [43,44]. The inhibition of CCL2 in animal models has been reported to modulate the inflammation cascade and ameliorate the symptoms of insulin resistance [36,45,46]. CCL2 has not been the subject of human studies on this matter, but it shows promise in both understanding the mechanisms behind the onset of IR and MS and in developing effective treatments for these conditions.

CCL4 (MIP-1β): Our results showed that mean levels of CCL4 were similar in male (186 ± 22.56 pg/mL) and female (194 ± 22.54 pg/mL) controls. CCL4 levels in T2DM subjects with normal body weight (Group A) and in overweight subjects (Group B) were significantly higher than in controls of both genders, with *p* < 0.05. T2DM subjects of both genders in Groups C and D (obese and severely obese) showed very highly significant elevations in CCL4 levels, with *p* < 0.001. In both genders, severely obese T2DM subjects (Group D) showed higher levels of CCL4 compared to obese Group C T2DM subjects with *p* < 0.04, and significantly elevated levels of CCL4 compared to Groups A and B with *p* < 0.01, as is displayed in Table 4 and Figure 5. Our results on CCL 4 are mostly consistent with previous studies, which reported higher levels of CCL4 in all types of diabetes, although there was no segregation of the subjects on the basis of gender or BMI [22,46,47]. CCL4 is a pro-inflammatory chemokine involved in the upregulation of the inflammatory pathways leading to IR, and its inhibition has been seen to improve IR and glycemic control in animal models [13,22]. The exact mechanism involving CCL4 in IR and MS and its possible therapeutic roles (if any) are still elusive and need further study.

CCL5 (RANTES): CCL4 exerts its effects by binding to the chemokine receptor CCR5 and is a pro-inflammatory chemokine [22]. Our study depicted similar serum levels of CCL5 in both the male and female control groups, as is true of other chemokines. In line with previous studies, our results showed a general trend of CCL5 elevation in T2DM subjects compared to the controls (irrespective of gender), but an increase in serum CCL5 in Group A T2DM subjects with normal body weight was highly pronounced with *p* < 0.001, in contrast to Groups B to D, as can be seen in Figure 4 and Figure 6 [22,36,41]. CCL5 helps in the recruitment of leucocytes to sites of inflammation and has been implicated in multiple disease conditions, including T2DM [30,34,35]. Our observation that T2DM subjects with varying levels of obesity (Groups B to D) showed significantly lower CCL5 levels, irrespective of gender, as compared to T2DM subjects with normal body weight (Group A), is somewhat bewildering and we do not have any concrete explanation for this phenomenon. It could be an observation that was overlooked in previous studies with no subgrouping on the basis of BMI in T2DM [22,43]. One possible explanation is that there might be some counterbalancing mechanism in overweight and obese T2DM subjects that leads to the underexpression of CCL5. This phenomenon needs to be explored in detail in future studies.

## 5. Limitations

This study might have some flaws because the sample size was not very big and we did not look at individual confounding factors like age and BMI. To find out what roles the chemokines listed above, and others, may play in the development of T2DM, future prospective studies with a large sample size are needed, and should take into account factors like age and BMI, as well as any other potential confounding factors.

## 6. Conclusions

The main findings of this investigation are summarized as follows:

- We observed that the mean serum concentrations of pro-inflammatory chemokines CCL 1, 2, 4, and 5 did not significantly differ between male and female control subjects.
- The concentrations of chemokines CCL1, CCL2, and CCL4 in the serum of T2DM subjects who had a normal body weight and those who were overweight were shown to be considerably elevated compared to the control group, regardless of gender. The observed increase demonstrated the greatest level of statistical significance among T2DM subjects with both obesity and severe obesity.
- The observation of a progressive rise in the blood concentrations of three pro-inflammatory chemokines (CCL1, 2, and 4) among T2DM subjects in relation to increasing BMI reinforces the notion that dyslipidemia and obesity play a substantial role in the pathogenesis of chronic inflammation, ultimately leading to insulin resistance.
- The levels of serum CCL5 exhibited a substantial and statistically significant increase across all groups of subjects with T2DM. However, this elevation was particularly prominent in individuals with a normal body weight.

Further work is warranted to explore potential counterbalancing mechanisms that may account for the observed underexpression of CCL5 in overweight and obese T2DM individuals.

## Figures and Tables

**Table 1:** Anthropometric indices and biochemical parameters of T2DM subjects and controls (male group).

	Controls	Group A	Group B	Group C	Group D
Anthropometric and biochemical indices	N = 96				
	N = 44	25	23	24	24
Age	46 (28–62)	43 (27–51)	47 (29–55)	49 (30–66)	47 (32–67)
WHR	0.86 (0.84–0.95)	0.92 (0.84–0.1.05)	0.94 (0.83–1.06)	1.07 **^T^ (0.97–1.11)	1.14 *^S^ (1.02–1.19)
BMI (kg/m^2^)	21.78 ± 1.78	21.80 ± 2.14	27.44 ± 2.29	34.94 ± 3.18 *^T^	47.22 ± 5.70 *^WX^
Fasting Glucose (mg/dL)	92 (78–116)	115 (88–130)	114 (92–138)	116 (92–144)	124 (103–152)
HbA1c (g/dL)	4.9 ± 0.88	7.4 ±1.02 *	8.1 ± 1.2 *	8.7 ± 1.48 *^S^	7.6 ± 0.78 *
Cholesterol-T (mg/dL)	186 (135–224)	204 ** (154–230)	214 * (148–232)	215 * (142–240)	226 * (158–258)
HDL-C (mg/dL)	53 (38–63)	46 (36–60)	48 (38–56)	45 (33–58)	44 (28–54)
LDL-C (mg/dL)	94 ± 32.20	98 (62–131)	118 ** (80–136)	120 ** (80–152)	136 *^w^ (95–162)
TG (mg/dL)	116 (86–132)	122 (96–136)	146 (98–178)	232 * (162–256)	242 *^w^ (162–286)

Age is presented in years with the range in parentheses. WHR—waist-to-hip ratio; BMI—body mass index; HbA1c—glycated hemoglobin; Cholesterol-T—total cholesterol; HDL—high-density lipoprotein; LDL—low-density lipoprotein; TG—triglycerides. Group A—T2DM subjects with normal body weight; Group B—overweight T2DM subjects; Group C—T2DM subjects with obesity; and Group D—T2DM subjects with severe obesity. * *p* < 0.001 vs. control group; ** *p* < 0.01 vs. control group; ^S^ *p* < 0.001 vs. Group A and B; ^T^ *p* < 0.01 vs. Group A and B; ^W^ *p* < 0.001 vs. controls and Group A; ^X^ *p* < 0.01 vs. Group C.

**Table 2:** Anthropometric indices and biochemical parameters of T2DM subjects and controls (female group).

	Controls	Group A	Group B	Group C	Group D
Anthropometric and biochemical indices	N = 74				
	N = 41	18	19	20	17
Age	48 (27–64)	44 (26–55)	47 (27–57)	46 (28–62)	48 (30–69)
WHR	0.85 (0.78–0.96)	0.91 (0.82–1.04)	0.92 (0.84–1.05)	1.06 **^T^ (0.97–1.13)	1.10 *^S^ (1.02–1.22)
BMI (kg/m^2^)	21.2 ± 1.66	21.41 ± 2.14	28.22 ± 2.26	33.88 ± 3.20 *^T^	46.48 ± 5.20 *^WX^
Fasting Glucose (mg/dL)	96 (82–114)	118 ** (90–141)	114 ** (92–138)	116 ** (92–144)	124 * (103–152)
HbA1c (g/dL)	4.5 ± 0.43	7.8 ± 1.02 *	8.1 ± 1.2 *	8.5 ± 1.48 *^S^	8.4 ± 0.98 *
Cholesterol-T (mg/dL)	168 (132–224)	196 ** (145–227)	204 ** (144–238)	215 ** (155–235)	228 ** (162–262)
HDL-C (mg/dL)	56 (41–66)	54 (39—62)	52 (40–63)	46 (35–64)	44 (35–62)
LDL-C (mg/dL)	96 ± 28.22	92 ± 28.28	111 ** ± 33.08	108 ** ± 30.30	132 * ± 32.12
TG (mg/dL)	96 (88–124)	126 ** (94–168)	142 ** (88–222)	152 ** (99–223)	195 *^WX^ (98–245)

Age is presented in years with the range in parentheses. WHR—waist-to-hip ratio; BMI—body mass index; HbA1c—glycated hemoglobin; Cholesterol-T—total cholesterol; HDL—high-density lipoprotein; LDL—low-density lipoprotein; TG—triglycerides; Group A—T2DM subjects with normal body weight; Group B—overweight T2DM subjects; Group C—T2DM subjects with obesity; and Group D—T2DM subjects with severe obesity. * *p* < 0.001 vs. control group; ** *p* < 0.01 vs. control group; ^S^ *p* < 0.001 vs. Groups A and B; ^T^ *p* < 0.01 vs. Groups A and B; ^W^ *p* < 0.001 vs. controls and Group A; ^X^ *p* < 0.01 vs. Group C.

**Table 3:** Summary of anthropometric indices, biochemical parameters, and chemokine levels in control and T2DM subjects.

Control: Female (N = 41)							Control: Male (N = 44)					
Name	Mean	SD	Median	Min	Max	SE	Mean	SD	Median	Min	Max	SE
Group	1.00	0.00	1.00	1.00	1.00	0.00	1.00	0.00	1.00	1.00	1.00	0.00
Age	47.73	11.55	50.00	27.00	64.00	1.80	45.34	9.99	45.50	25.00	62.00	1.51
Sex	1.00	0.00	1.00	1.00	1.00	0.00	2.00	0.00	2.00	2.00	2.00	0.00
WHR	0.87	0.06	0.86	0.78	0.98	0.01	0.90	0.03	0.91	0.86	0.96	0.00
BMI	20.93	1.21	20.55	19.23	22.92	0.19	21.98	1.26	22.26	19.89	23.54	0.19
Glu-F	96.88	9.09	98.00	82.00	114.00	1.42	96.25	10.27	96.00	78.00	115.00	1.55
HbA1c	4.49	0.31	4.39	4.10	4.94	0.05	5.03	0.47	5.02	4.12	5.66	0.07
Chol-T	182.37	23.89	180.00	136.00	224.00	3.73	177.75	25.57	178.00	135.00	216.00	3.85
HDL	49.41	9.59	48.00	35.00	66.00	1.50	49.95	6.24	51.50	35.00	62.00	0.94
LDL	96.20	14.84	97.00	68.00	125.00	2.32	101.23	14.68	98.50	78.00	126.00	2.21
TG	106.73	14.16	102.00	87.00	156.00	2.21	105.89	14.77	104.00	86.00	132.00	2.23
CCL1	154.51	12.70	156.00	130.00	174.00	1.98	157.73	13.64	156.50	135.00	178.00	2.06
CCL2	277.39	32.76	288.00	165.00	312.00	5.12	286.82	19.43	286.50	245.00	322.00	2.93
CCL4	196.76	11.60	199.00	172.00	216.00	1.81	187.30	15.63	188.50	148.00	210.00	2.36
CCL5	4804.98	185.02	4768.00	4414.00	5098.00	28.89	4914.45	218.42	4890.00	4530.00	5240.00	32.93
T2DM Group: Female (N = 74)							T2DM: Male (N = 96)					
Name	Mean	SD	Median	Min	Max	SE	Mean	SD	Median	Min	Max	SE
Group	2.00	0.00	2.00	2.00	2.00	0.00	2.00	0.00	2.00	2.00	2.00	0.00
Age	44.50	9.90	43.00	25.00	66.00	1.15	45.26	9.86	45.00	28.00	67.00	1.01
Sex	1.00	0.00	1.00	1.00	1.00	0.00	2.00	0.00	2.00	2.00	2.00	0.00
WHR	1.00	0.11	0.98	0.82	1.22	0.01	1.03	0.09	1.02	0.84	1.19	0.01
BMI	31.74	9.36	29.54	19.56	51.70	1.09	36.54	10.79	38.25	21.80	52.90	1.10
Glu.F	121.57	20.97	126.00	86.00	156.00	2.44	124.26	22.04	129.00	45.00	156.00	2.25
HbA1c	7.79	0.90	7.56	6.68	9.42	0.10	8.15	1.18	7.92	6.08	10.22	0.12
Chol.T	205.57	37.87	202.00	134.00	294.00	4.40	218.20	32.22	226.50	154.00	258.00	3.29
HDL	47.32	9.01	48.00	6.00	64.00	1.05	44.64	6.13	45.00	34.00	56.00	0.63
LDL	121.99	27.13	125.50	70.00	164.00	3.15	125.16	26.93	134.00	62.00	162.00	2.75
TG	157.81	55.60	156.00	87.00	254.00	6.46	196.64	55.59	207.00	96.00	288.00	5.67
CCL1	309.72	62.47	288.50	230.00	424.00	7.26	338.50	72.10	367.00	216.00	426.00	7.36
CCL2	365.59	53.18	357.50	288.00	462.00	6.18	393.90	50.30	398.00	232.00	467.00	5.13
CCL4	281.12	41.45	272.00	218.00	358.00	4.82	292.08	57.09	294.00	202.00	552.00	5.83
CCL5	7678.62	1194.52	7126.50	6480.00	10,068.00	138.86	7546.95	1008.62	7250.00	6312.00	9200.00	102.94

**Table 4:** The serum levels of chemokines CCL1, CCL2, CCL4, and CCL5 in male T2DM subjects and control subjects.

Subject Groups	CCL1 (pg/mL)	CCL2 (pg/mL)	CCL4 (pg/mL)	CCL5 (pg/mL)
Controls (N = 44)	158 ± 21.02	288 ± 33.12	186 ± 22.56	4880 ± 348.98
A (N = 25)	245 ± 27.46 **	332 ± 35.76 ***	232 ± 25.44 ***	9620 ± 523.22 *
B (N = 23)	274 ± 23.12 **	344 ± 35.87 ***	245 ± 25.72 ***	7266 ± 387.55 **^N^
C (N = 24)	326 ± 35.44 *^N^	376 ± 36.92 *^N^	290 ± 24.14 *^O^	7056 ± 382.34 **^N^
D (N = 24)	388 ± 37.12 *^LM^	422 ± 44.25 *^LM^	332 ± 29.90 *^LM^	6892 ± 377.16 ***^NQ^

Group A—T2DM subjects with normal body weight; Group B—overweight T2DM subjects; Group C—T2DM subjects with obesity; and Group D—T2DM subjects with severe obesity. * *p* < 0.001 vs. control group; ** *p* < 0.01 vs. control group; *** *p* < 0.05 vs. control group; ^L^ *p* < 0.04 vs. Group C; ^M^ *p* < 0.01 compared to Groups A and B; ^N^ *p* < 0.01 vs. Group A; ^O^ *p* < 0.04 compared to Groups A and B; ^Q^ *p* < 0.01 compared to Group A.

**Table 5:** The serum levels of chemokines CCL1, CCL2, CCL4, and CCL5 in female T2DM subjects and control subjects.

Subject Groups	CCL1 (pg/mL)	CCL2 (pg/mL)	CCL4 (pg/mL)	CCL5 (pg/mL)
Controls (N = 41)	152 ± 20.88	275 ± 32.14	194 ± 22.54	4756 ± 342.56
A (N = 18)	262 ± 27.52 **	328 ± 35.66 ***	244 ± 25.38 ***	9546 ± 522.44 *
B (N = 19)	311 ± 25.10 **	348 ± 35.58 ***	248 ± 25.24 ***	7168 ± 388.24 **^N^
C (N = 20)	318 ± 35.08 *^N^	382 ± 36.82 *^N^	296 ± 26.08 *^O^	6978 ± 384.74 **^N^
D (N = 17)	374 ± 37.04 *^LM^	418 ± 44.16 *^LM^	328 ± 29.88 *^LM^	6866 ± 376.12 ***^NQ^

Group A—T2DM subjects with normal body weight; Group B—overweight T2DM subjects; Group C—T2DM subjects with obesity; and Group D—T2DM subjects with severe obesity. * *p* < 0.001 vs. control group; ** *p* < 0.01 vs. control group; *** *p* < 0.05 vs. control group; ^L^ *p* < 0.04 vs. Group C; ^M^ *p* < 0.01 compared to Groups A and B; ^N^ *p* < 0.01 vs. Group A; ^O^ *p* < 0.04 compared to Groups A and B; ^Q^ *p* < 0.01 compared to Group A.

## Data Availability

The data will be made available on a reasonable request.
